# Strong association of tissue inhibitor of metalloproteinase (TIMP)-2 and -3 promoter single nucleotide polymorphisms with risk of colorectal cancer in ethnic Kashmiri population – a case control study

**DOI:** 10.1042/BSR20190478

**Published:** 2019-05-10

**Authors:** Mujeeb Zafar Banday, Aga Syed Sameer

**Affiliations:** 1 Department of Biotechnology, University of Kashmir, Hazratbal, Srinagar, Kashmir, India; 2 Department of Biology, United Arab Emirates University (UAEU), Al Ain, Abu Dhabi, United Arab Emirates; 3 Department of Basic Medical Sciences, College of Medicine, King Saud Bin Abdulaziz University for Health Sciences, Jeddah, Saudi Arabia; 4 King Abdullah International Medical Research Centre (KAIMRC), Jeddah, Saudi Arabia

**Keywords:** Case control study, Colorectal cancer (CRC), Kashmir, tissue inhibitors of metalloproteinases (TIMPs

## Abstract

**Background:** The tissue inhibitors of metalloproteinases (TIMPs) including TIMP2 and TIMP3 are the key physiological inhibitors of matrix metalloproteinases (MMPs) and along with MMPs, TIMPs play a vital role in the coordinated proteolytic breakdown and remodeling of the extracellular matrix (ECM) and the basement membrane that represent the barriers to any malignant tumor invasion and progression. These enzymes are vital for tumor invasion and metastasis and also play a critical role in several other stages of tumor development and progression. The studies on the association of various polymorphisms in human TIMP2 and TIMP3 genes including TIMP2-418G/C and TIMP3-1296T/C single nucleotide polymorphisms (SNPs) and CRC risk are limited, mixed, and inconclusive.

**Materials and methods:** The aim of the present study was to analyze the association of TIMP2-418G/C and TIMP3-1296T/C promoter SNPs with colorectal cancer (CRC) susceptibility and development risk and also to evaluate the modifying effects of possible TIMP2-418G/C and TIMP3-1296T/C SNPs’ genotypes on different risk factors of CRC or the reciprocal effect in ethnic population of Kashmir, India through a case–control setup. The genotype frequencies of TIMP2-418G/C and TIMP3-1296T/C promoter SNPs were compared between 142 CRC patients and 184 individually matched healthy controls by using polymerase chain reaction-restriction fragment length polymorphism (PCR-RFLP) method. The associations between the TIMP2-418G/C and TIMP3-1296T/C SNPs and CRC risk were examined through conditional logistic regression models adjusted for multiple possible confounding (third) variables. The possible effect measure modification of the association between the relevant SNP genotypes and CRC risk by various CRC risk factors including age, gender, and smoking status was also analyzed. Further, the associations between these SNPs and various clinico-pathological parameters, demographic variables, and environmental factors within the case group subjects with regard to CRC risk were also evaluated.

**Results:** The overall association between the TIMP2-418G/C and TIMP3-1296T/C SNPs and the modulation of CRC risk was found to be highly significant (*P*=0.019 and *P*=0.000 for TIMP2 and TIMP3 SNPs, respectively). The heterozygous genotype (GC) of TIMP2-418G/C was significantly associated with an increased risk of colorectal cancer [OR, 1.87 (95%CI, 1.07–3.27); *P*=0.027] whereas the heterozygous genotype (TC) of TIMP3-1296T/C SNP was significantly associated with a decreased risk of colorectal cancer [OR, 0.53 (95%CI, 0.32–0.86); *P*=0.011]. The variant genotype (CC) of TIMP3-1296T/C SNP was also significantly associated with a decreased risk of colorectal cancer [OR, 0.18 (95%CI, 0.05–0.65); *P*=0.009].

**Conclusion:** The present study demonstrates that there is a strong and highly significant association between the TIMP2-418G/C and TIMP3-1296T/C promoter SNPs and the risk of developing CRC in ethnic Kashmiri population. However, in order to substantiate our findings, the present study needs to be replicated with bigger sample size and should involve other ethnically defined populations with high CRC risk.

## Introduction

Colorectal cancer (CRC) defined as the neoplasia of the colon, rectum, and appendix is the third most common cause of cancer-related morbidity and mortality worldwide with nearly 1.2 million new cases diagnosed each year and approximately 600,000 deaths reported annually [1,2]. The Valley of Kashmir, part of Jammu and Kashmir State, located in Northern India is inhabited by unique ethnic population and here CRC represents the third most common gastrointestinal (GIT) cancer [3] and is the fourth frequent cancer among the males and the third among the females [3,4].

The invasiveness and the metastatic ability of colorectal tumors to invade tissue, vascular and lymphatic vessels are mainly mediated through the coordinated proteolytic breakdown and remodeling of the extracellular matrix (ECM) including the basement membrane that represents the barriers to any malignant tumor invasion and progression. Matrix metalloproteinases (MMPs), a family of zinc-dependent endopeptidases and their physiological inhibitors that regulate the activity of MMPs, in particular, the tissue inhibitors of metalloproteinases (TIMPs) are key players in the coordinated proteolytic breakdown and remodeling of the ECM and the basement membrane which besides making them important for several physiological processes also makes them vital for tumor invasion and metastasis [5,6]. Besides, they also play a critical role in several other stages of tumor development and progression. The altered TIMP expression and activity coupled with increased expression and activity of MMPs have been described in almost every type of human cancer including colorectal cancer [5,7].

The TIMPs are natural highly specific endogenous inhibitors of MMPs. Human TIMP family consists of four 21–28 kDa proteins known as TIMP1, TIMP2, TIMP3, and TIMP4 encoded by four paralogous genes [8]. TIMPs are vital to the maintenance of ECM homeostasis primarily through their MMP inhibitory functions. The maintenance of critical physiological equilibrium between MMPs and TIMPs is essential for normal cellular functioning. However, in addition to the MMP inhibitory activities, TIMPs play essential roles in many physiological processes including modulation of cell proliferation, migration and invasion and synaptic plasticity besides their anti-angiogenesis and both anti- and pro-apoptotic activities [9,10].

TIMPs play vital role in several physiological processes occurring in the human gut. They are particularly important in regulating the gut inflammation and are involved in several pathophysiological processes in the gut that are associated with an increased predisposition to CRC [11]. The uncontrolled MMP activity resulting from reduced inhibition of MMPs has been associated with various pathophysiological conditions including neoplastic diseases. The disturbance of the physiological equilibrium between MMPs and TIMPs, which leads to altered ECM homeostasis, is a critical event in facilitating tumor initiation, early phases of tumor progression and the distant metastases in most of the cancers including CRC. TIMPs influence tumor progression and metastasis through the inhibition of MMPs and through direct modulation of angiogenesis and apoptosis [5,10,12].

TIMP2 is a highly potent MMP inhibitor capable of inhibiting all MMPs particularly gelatinases, MMP2 and MMP9, is constitutively expressed in contrast with other TIMPs and MMPs which are inducible except MMP2 and is an important mediator in the activation of ubiquitously expressed pro-MMP2 and increased expression of MMP2 has been associated with tumor growth and invasion [13]. The expression profile of TIMP2 has been studied in colorectal cancer. The reduced expression and activity of TIMP2 has been associated with increased invasiveness, advancing tumor stage, poor prognosis, and decreased overall survival in CRC [14,15]. The reduced TIMP2 expression which translates into increased MMP2/TIMP2 ratio has been reported in colorectal cancer [16]. The increased MMP2/TIMP2 ratio has been reported to exhibit direct correlation with advanced tumor stage [17], increased colorectal tumor invasion [15,18] and poor prognosis [14]. Further, increased TIMP2 expression has been reported to be associated with an increased overall survival [18].

Another member of this family, TIMP3 in addition to MMPs also inhibits several members of the ADAM and ADAMTS families including TNF-α converting enzyme (TACE/ADAM17) and the aggrecanases, ADAMTS-4, and ADAMTS-5 [19]. Further, TIMP3 unlike others TIMPs is not secreted but remains attached to the ECM through its N- and the C-terminal domains [8,19]. The decreased expression and activity of TIMP3 has been reported to correlate directly with an increased CRC invasion [20] and advanced tumor stage [21]. TIMP3 has been reported to induce apoptosis and inhibit growth and proliferation in certain colon cancer cell lines [20]. Further, the increased TIMP3 expression and activity resulted in decreased tumor growth and liver metastasis in CRC and *in vitro* studies have shown that this increased expression and activity reduced tumor invasiveness and metastasis by decreasing or inhibiting pro-tumorigenic cell adhesion and migration [22].

The TIMP2 gene is located on the long arm of chromosome 17 at position 25.3 (17q25.3), and TIMP3 gene is located on the long arm of chromosome 22 at position 12.3 (22q12.3). The expression of TIMPs is tightly regulated at the transcriptional level and involves the role of various soluble factors including cytokines and growth factors. Several functional single nucleotide polymorphisms (SNPs) within the promoter regions of human TIMP genes have been reported to affect the production of the TIMPs in question through their effect on transcriptional activity that in turn leads to altered gene expression, secretion, and activity of these TIMPs and confer differences in susceptibility between different individuals to several diseases including various cancers. Among these polymorphisms, the two functional SNPs located in the promoter region which have pivotal allele-specific effects on the regulation of TIMP2 and TIMP3 gene transcription and consequently the TIMP2 and TIMP3 protein activity occur at positions −418 and −1296 nucleotides (nt) relative to the transcription start site respectively representing a G to C transition and designated as TIMP2-418G/C SNP (rs8179090) [23] and representing a T to C transition and designated as TIMP3-1296T/C SNP (rs9619311) [24] are the most significant.

The TIMP2-418G/C SNP has been previously reported to be associated with risk modulation in colorectal cancer [14] and other cancers including breast cancer [25], gastric cancer [26]; oral cancer [27], prostate cancer [28], ovarian cancer [29], head and neck cancer [23], and non-Hodgkin’s lymphoma [30]. The association of TIMP3-1296T/C SNP has been reported in breast cancer [25] gastroesophageal adenocarcinoma [31] and hepatocellular carcinoma (HCC) [32]. We could not find any report on association studies between TIMP3-1296T/C SNP and CRC. Besides the studies on association of TIMP2-418G/C SNP with CRC were also scarce. In general, the association studies for these TIMP SNPs and cancer risk are sparse and the results are mixed. The present study could therefore provide a useful insight into the possible link between TIMP2-418G/C and TIMP3-1296T/C SNPs and CRC risk.

In the present study, we systematically evaluated the possible association between TIMP2-418G/C and TIMP3-1296T/C SNPs and susceptibility to colorectal cancer in Kashmiri population through a case–control setup. We analyzed the possible effect modification of CRC risk by age, gender, and smoking status. Further, we investigated the possible relationship of these SNPs with various clinico-pathological parameters, demographic variables, and environmental factors including smoking habit and evaluated their role in modulating the risk of colorectal cancer in the population under study.

## Participants and methods

### Study subjects

The present study involved two subject groups: case and control. The case group included 142 patients recruited consecutively irrespective of their age and gender with primary colorectal cancer who underwent surgical resection for primary CRC tumors at the Department of General Surgery, Sher-I-Kashmir Institute of Medical Sciences (SKIMS), Srinagar, Kashmir. The diagnosis of colorectal cancer was confirmed histopathologically. The tumor stage and the tumor grade were classified according to the eighth edition of TNM classification of Union International Control of Cancer (UICC). Only those cases who had not received any neoadjuvent chemo or radiotherapy were chosen for the present study. All the cases were more than 18 years old and had no prior history of any malignancy. Blood and tissue samples were obtained from these CRC patients. The control group included 184 healthy individuals with no history or prior diagnosis of any malignant disorder or any other serious disease that were recruited during the same time period and from the same geographic area and from whom blood was collected and used as control for the present study. The control group included both the general population-based subjects and hospital-based subjects. The control group subjects were matched to the case group subjects individually for age (±5 years), sex, place of residence (rural/urban), smoking habit and ethnicity in order to minimize the confounding effect of these various relevant factors. The inclusion and the exclusion criteria for the recruitment of case and control subjects were decided keeping in view all the factors that could affect the final outcome and the reliability of the study. The case subjects included in the present study were those with histopathologically confirmed CRC; were above 18 years old with no prior history of any malignancy and were ethnically homogenous Kashmiri population. The control subjects chosen for the present study were those with no history or prior diagnosis of any malignant disorder or any other serious disease; were above 18 years old. Both the case and the control subjects chosen for the present study were ethnic Kashmiris.

### Data collection

The data relevant to the study concerning all the CRC patients including various clinico-pathological parameters, demographic variables, and the environmental factors were obtained and evaluated from the patient medical records (files), pathology reports, and also from the personal interviews with the patients and/or their guardians (for those who were illiterate or unable to communicate). The interviews were conducted in local language for easy and direct communication, which also helped to gather maximum possible relevant information. The data collected included tumor location, Dukes Stage, lymph node status, age, sex, and place of residence, ethnicity, smoking habit, and the family history of cancer among several other potential confounding parameters. The relevant data were also obtained for each of the recruited controls mostly through personal interviews and included parameters like age, sex, place of residence, ethnicity, and smoking habit. The data collection was carried out by research professionals only in order to fulfill requisite quality standards during the course of the present study. All the patients and/or their guardians were informed about the study and their willingness to participate in the present study was documented using a predesigned questionnaire and same procedure was followed for the controls. All the procedures concerning the study subjects including sample procurement and the data collection were carried out in accordance with the ethical standards laid down by the Institutional Ethics Committee (IEC), SKIMS, and the World Health Organization (WHO) and the Code of Ethics of the World Medical Association (Declaration of Helsinki, 1964 and its seventh amendment, 2013) for experiments in humans [33].

### Sample preparation and DNA extraction

The tumor tissue samples collected after surgical resection were immediately snap frozen in liquid nitrogen and then stored at  −80°C until further use for DNA extraction and other experimental purposes. Prior to DNA extraction, tumor tissue samples were washed two to three times in phosphate buffered saline (PBS) and adipose and connective tissue portions if any were dissected away. Peripheral blood sample, 3–5 ml from each case and control group individual was collected by venopuncture into ethylene diamine tetra acetic acid (EDTA) coated blood vacutainer collection tubes (purple capped tubes; ADS Hitech Polymers, India) and stored at −80°C until further use. The genomic DNA was extracted from both the tumor tissue and blood specimens using DNeasy™ Blood and Tissue Kit (catalog no. 69504; Qiagen, Germany) and Quick-gDNA™ MiniPrep kit (catalog no. D3024; Zymo Research, U.S.A.) according to the manufacturers’ instructions. The extracted DNA was stored at −20°C until further use. The qualitative and the quantitative assessments of the extracted genomic DNA samples were carried out by absorbance measurements at 260 and 280 nm using UV-visible spectrophotometric analysis and also by agarose gel electrophoresis. The DNA extracted from blood samples of case and control group subjects was used for the present study.

### Single nucleotide polymorphism analysis or genotyping

The TIMP2-418G/C (rs8179090) and TIMP3-1296T/C (rs9619311) SNPs were genotyped using polymerase chain reaction-restriction fragment length polymorphism (PCR-RFLP) assay.

### TIMP2-418G/C and TIMP3-1296T/C PCR

The PCR for the amplification of gene regions encompassing the TIMP SNPs under study was carried out in a total volume of 25 µL containing 100 ng–1 µg of genomic DNA, 0.7–1 U Taq DNA polymerase with lX Standard Taq reaction buffer (New England Biolabs, U.K.), 1.68 mM MgCl_2_ for TIMP2-418G/C SNP and 1.8 mM MgCl_2_ for TIMP3-1296T/C SNP; 0.28 mM deoxynucleotide triphosphate mix (New England Biolabs, U.K.); 0.56 µM forward and reverse oligonucleotide primers (Integrated DNA Technologies, India) and nuclease–protease free water (Qiagen, Germany) added up to a final volume of 25 µL. Alternatively and randomly Phusion DNA Polymerase with Phusion HF Buffer (New England Biolabs, Inc. U.K.) was used instead of Taq DNA polymerase with Standard Taq reaction buffer to check for any Taq polymerase induced amplification errors.

**Figure 1:**
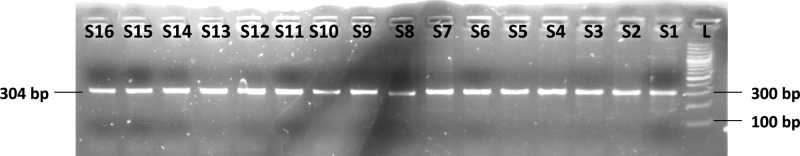
Electrophoresis of TIMP2-418G/C SNP PCR products on a 2.5% agarose gel *Lanes S1–S16*: Amplified PCR products with prominent/desired band 304 bp in size. *Lane L*: 100 bp molecular size marker/ladder.

**Figure 2:**
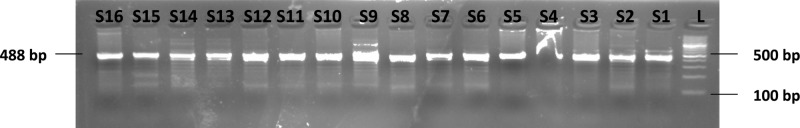
Electrophoresis of TIMP3-1296T/C SNP PCR products on a 2.5% agarose gel *Lanes S1–S16*: Amplified PCR products with prominent/desired band 488 bp in size. *Lane L*: 100 bp molecular size marker/ladder.

The PCR conditions used for TIMP amplification were as follows: initial denaturation at 95°C for 6 min followed by 35 cycles of denaturation at 95°C for 45 s; annealing at 61°C for 45 s for TIMP2-418G/C SNP and at 58°C for 60 s for TIMP3-1296T/C SNP and extension at 72°C for 45 s followed by a single final extension step at 72°C for 10 min. The oligonucleotide primers used for the amplification of the specific promoter region containing the TIMP2-418G/C SNP were 5′-CGTCTCTTGTTGGCTGGTCA-3′ (Forward) and 5′-CCTTCAGCTCGACTCTGGAG-3′ (Reverse) and oligonucleotide primers for TIMP3-1296T/C SNP were 5′-CAAAGCAGAATCAAGATGTCAAT-3′ (Forward) and 5′-CTGGGTTAAGCAACACAAAGC-3′ (Reverse). The desired PCR products obtained for TIMP2-418G/C and TIMP3-1296T/C SNPs were 304 and 488 bp in size, respectively (see Figures 1 and 2 for PCR gel pictures).

### Genotyping

**Figure 3:**
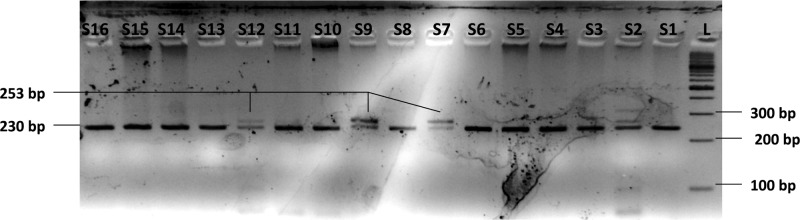
Electrophoresis of TIMP2-418G/C SNP genotyping by PCR-RFLP on a 4% agarose gel *Lanes S1–S16*: Restriction digestion products; wild genotype (GG) is cleaved by *Ava*I enzyme yielding three fragments of size 230, 51, and 23 bp whereas the variant genotype (CC) yields two fragments, 253 and 51 bp in size. Heterozygous genotype (GC) yields four fragments 253, 230, 51, and 23 bp in size. The 51 and 23 bp fragments are not visible in the picture. *Lanes S2, S7, S9, and S12* show the heterozygous genotype (GC) whereas rest of the lanes show the wild genotype (GG) of TIMP2-418G/C SNP. Variant genotype (CC) of TIMP2-418G/C SNP was not observed in any of the samples studied. *Lane L*: 100 bp molecular size marker/ladder.

**Figure 4:**
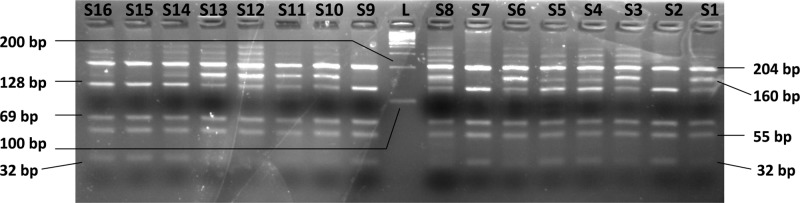
Electrophoresis of TIMP3-1296T/C SNP genotyping by PCR-RFLP on a 4% agarose gel *Lanes S1–S16*: Restriction digestion products; wild genotype (TT) is cleaved by *Alu*I enzyme yielding five fragments of size 204, 128, 69, 55, and 32 bp whereas the variant genotype (CC) yields four fragments of size 204, 160, 69, and 55 bp. Heterozygous genotype (TC) yields six fragments 204, 160, 128, 69, 55, and 32 bp in size. *Lanes S2, S7, S9, and S15* show the wild genotype (TT) whereas the rest of the lanes show the heterozygous genotype (TC) of TIMP3-1296T/C SNP. The variant genotype (CC) is not represented in this picture. *Lane L*: 100 bp molecular size marker/ladder.

The TIMP2-418G/C and TIMP3-1296T/C SNPs were genotyped using the restriction enzymes *Ava*I (*Bso*B1, *Eco*88I) and *Alu*I, respectively. Both these restriction enzymes were procured from Thermo Fisher Scientific, U.S.A. The digestion was carried out according to the manufacturers’ instructions in a 30 µL reaction volume containing 10 µL of PCR product and 10 U of appropriate restriction enzyme and incubated at 37°C overnight. The digestion products of TIMP2-418G/C and TIMP3-1296T/C SNPs were separated on 4% agarose gels stained with ethidium bromide (HiMedia, India) to a final concentration of 0.5 µg/ml. The genotyping information including fragment lengths of digested products (see Figures 3 and 4 for RFLP digestion gel pictures), enzyme recognition sequence, and other important parameters are summarized in Table 1.

**Table 1:** Genotyping information of TIMP2-418G/C and TIMP3-1296T/C SNPs

Gene-SNP name	Ref SNP ID	Restriction enzyme	Recognition sequence^*^	Wild type fragment length	Variant (mutant) type fragment length	Heterozygous type fragment length
TIMP2-418G/C	rs8179090	*Ava*I (*Bso*B1, *Eco*88I)	5′-C|YCGRG-3′, 3′-GRGCY|C-5′	230, 51, and 23 bp (GG)	253 and 51 bp (CC)	253, 230, 51, and 23 bp (GC)
TIMP3-296T/C	rs9619311	*Alu*I	5′-AG|CT-3′, 3′-TC|GA-5′	204, 128, 69, 55, and 32 bp (TT)	204, 160, 69, and 55 bp (CC)	204,160, 128, 69, 55, and 32 bp (TC)

* (|) refers to the point where the restriction enzyme cleaves the sequence.

### Quality control

The quality control involved the assessment of genotyping errors including the false estimates of a particular allele frequency and the evaluation of the reproducibility of the genotyping done. For these assessments, approximately 10% of the case and control samples selected randomly were re-genotyped. In addition, in each PCR-RFLP setup, previously amplified and genotyped samples representing different genotypic scenarios were included as a reference control. The genotyping reproducibility was very high for both the SNPs studied with a weighted kappa coefficient of 0.99 for TIMP2-418G/C SNP and 0.97 for TIMP3-1296T/C SNP which meant a high concordance rate of 99% and 97% for TIMP2-418G/C SNP and TIMP3-1296T/C SNP, respectively [34,35].

### Statistical analyses

The frequencies of genotypes and alleles for the SNPs under study were obtained through direct counting. The numbers and percentages were calculated and presented for each of the categorical variables along with means, standard deviations (SD), median, and inter-quartile range for continuous variables. Conditional logistic regression analysis was carried out to calculate unadjusted and adjusted odds ratios (ORs) and corresponding 95% confidence intervals (CIs) to assess the possible association of the relevant SNPs’ genotypes with CRC risk and to assess the possible gene–environment interactions if applicable. The more common homozygous genotype and/or more common haplotype was used as the baseline or reference group in the conditional logistic regression model and the genotypes in order of more common/wild homozygous, heterozygous and less common/variant homozygous were, respectively, represented by the ordinal values 1, 2, and 3 during the statistical analyses. In order to eliminate the possible confounding (third) variables, the conditional logistic regression models were adjusted for the known CRC risk factors like gender, age, and smoking habit and also with the place of residence. The possible effect measure modification or effect modification of the association between various genotypes relevant to the SNPs under study and CRC risk by various risk factors including age, gender, and smoking status was also analyzed using conditional logistic regression. The correlation between the genotypes and the clinico-pathological parameters, demographic variables and environmental factors including smoking habit within the case group was analyzed by Fisher exact test. The fitness of the genotype distributions to Hardy–Weinberg equilibrium (HWE) for the SNP allele and the genotype frequencies in the population under study was tested using the chi-square test. A two-sided probability value of or less than 5% (*P*≤0.05) was considered statistically significant for all types of analyses. All statistical analyses were performed using IBM SPSS Statistics v21 software.

The effective sample size and the statistical power were computed using the ‘Genetic Power Calculator’ developed by Purcell et al. (http://zzz.bwh.harvard.edu/gpc/). The statistical power of 80% is widely used in genetic association studies to avoid Type II errors and to determine a cost-effective sample size under the assumption of 10–25% variant allele frequency, 1:1 case-to-control ratio, and 5% Type I error rate (*α*). We obtained a healthy power score of more than 85% for both the SNPs under study in our case–control study design with 142 case subjects and 184 control subjects.

## Results

### General characteristics of study subjects

The present study included a total of 142 primary colorectal cancer patients and 184 control subjects with prior consent of each individual. The rate of participation was 100% for both the study groups because only those study subjects were considered who fully agreed to participate in the present study. This was done to ensure exhaustive data collection relevant to the study concerning all the CRC patients and control subjects. The frequencies of various clinico-pathological parameters, demographic variables and environmental factors in colorectal cancer case subjects and relevant parameters in control subjects are given in Table 2. The mean age of the case group subjects was 52.68 years; for males, it was 54.70 years and for females it was 50.66 years and the age range was 21–82 years. Further, 53.52% (76/142) of case group subjects were >50 years old and 46.48% (66/142) subjects were ≤50 years of age. This group consisted of 59.86% (85/142) males and 40.14% (57/142) females (male/female ratio = 1.49). The mean age of the control group subjects was 52.22 years; for males, it was 53.64 years and for females it was 50.80 years and the age range was 21–80 years. Further, 50.54% (93/184) subjects were >50 years old and 49.46% (91/184) subjects were ≤50 years of age. The control group consisted of 55.43% (102/184) males and 44.57% (82/184) females (male/female ratio = 1.24). The difference in the distribution of gender and age among the case group and control group subjects was not statistically significant (*P*>0.05) (Table 2). The case group subjects consisted of 61.27% (87/142) rural residents and 38.73% (55/142) urban residents and the control group consisted of 54.89% (101/184) rural residents and 45.11% (83/184) urban residents. Further, the case group consisted of 56.34% (80/142) smokers and 43.66% (62/142) non-smokers while as the control group consisted of 51.09% (94/184) smokers and 48.91% (90/184) non-smokers. No statistically significant dwelling and smoking status related differences were observed between the case and control group subjects (*P*>0.05) (Table 2).

**Table 2:** General characteristics of study subjects

Characteristics	Colorectal cancer cases (*N* = 142)^*^	Controls (*N* = 184)^*^	Pearson *χ*^2^; *P* value
**Age (years)**			
Mean age (SD) (SEM)^†^	52.68 (15.34) (1.29)	52.22 (14.57) (1.07)	
Age range (Median)	21–82 (55)	21–80 (51.5)	
≤50	66 (46.48%)	91 (49.46%)	0.29; 0.59
>50	76 (53.52%)	93 (50.54%)	
**Gender**			
Male	85 (59.86%)	102 (55.43%)	0.64; 0.42
Female	57 (40.14%)	82 (44.57%)	
**Place of residence**			
Rural	87 (61.27%)	101 (54.89%)	1.33; 0.25
Urban	55 (38.73%)	83 (45.11%)	
**Smoking status**			
Ever	80 (56.34%)	94 (51.09%)	0.89; 0.35
Never	62 (43.66%)	90 (48.91%)	
**Tumor location**			
Colon	58 (40.85%)		
Rectum	84 (59.15%)		
**Tumor grade**			
W.D.	95 (66.90%)		
M.D. and P.D.	47 (33.10%)		
**Lymph node status**			
Involved	78 (54.93%)		
Not Involved	64 (45.07%)		

Various clinico-pathological parameters, demographic variables, and environmental factors in colorectal cancer case subjects and relevant parameters in control subjects from Kashmir.

* *N* denotes number of subjects or individuals.

† SD and SEM stand for standard deviation and standard error of mean, respectively. Pearson chi-square test (*χ*^2^) was used to calculate the *P* values for categorical variables.

### Association analysis of TIMP2-418G/C (rs8179090) SNP

The frequencies of the genotypes of TIMP2-418G/C SNP for both the case and the control groups are listed in Table 3. The more common GG genotype of TIMP2-418G/C SNP was higher in frequency among the case group [82.40% (117/142)] in comparison with the control group [71.20% (131/184)]. The frequency of the heterozygous genotype (GC) in the case group [17.60% (25/142)] was lower than that of the control group [28.80% (53/184)]. The TIMP2-418G/C SNP variant genotype (CC) was not found in either of the two study groups (case and control). Further, the frequency of the more common TIMP2-418G allele was found to be 91.20% (259/284) among the case group subjects and 85.60% (315/368) among the control group subjects. The frequency of the less common TIMP2-418C allele was found to be 8.80% (25/284) among the case group subjects and 14.40% (53/368) among the control group subjects (Table 3). The frequency of the combined variant genotype (GC + CC) in the case group [17.60% (25/142)] was less than that of the control group [28.80% (53/184)]. The overall association between the TIMP2-418G/CSNP and the modulation of colorectal cancer risk was found to be significant (*P*=0.019) (Table 3). The heterozygous genotype (GC) was significantly associated with an increased risk of colorectal cancer [OR, 1.87 (95%CI, 1.07–3.27); *P*=0.027]. Further, the less common TIMP2-418C allele showed an overall significant association with colorectal cancer (*P*=0.029). The TIMP2-418C allele was associated with an increased risk of the colorectal cancer [OR, 1.74 (95%CI, 1.05–2.88); *P*=0.029]. The combined variant genotype (GC + CC) also showed an overall significant association with colorectal cancer (*P*=0.019). Further, the genotype frequencies for TIMP2-418G/C SNP among the case group subjects was found to be in agreement with HWE (*χ*^2^ = 1.323; *P*=0.25) but not among the control group subjects (*χ*^2^ = 5.21; *P*=0.022).

**Table 3:** TIMP2-418G/C SNP genotype frequency distributions among CRC cases and matched controls and risk of CRC^*^

	CRC cases (*N* = 142)^*^	Controls (*N* = 184)^*^	OR (95%CI); *P* value^†^	Adjusted OR^‡^ (95% CI); *P* value^†^	*χ*^2^; Pearson *P* value (overall)^†^^,^^§^
**Genotype**					
GG	117(82.40%)	131(71.20%)	**1.0 (Reference)**	**1.0 (Reference)**	5.52; **0.019**
GC	25(17.60%)	53(28.80%)	**1.87 (1.07–3.25); 0.027**	**1.87 (1.07–3.27); 0.027**	
CC	0(0%)	0(0%)	–	–	
GC + CC	25(17.60%)	53(28.80%)	**1.87 (1.07–3.25); 0.027**	**1.87 (1.07–3.27); 0.027**	5.52; **0.019**
**Allele**					
G	259(91.20%)	315(85.60%)	**1.0 (Reference)**		
C	25(8.80%)	53(14.40%)	**1.74 (1.05–2.88); 0.029**		4.77; **0.029**

* *N* denotes number of subjects or individuals.

† The values in bold indicate significant results. ORs (95% CIs) were obtained from conditional logistic regression models.

‡ Adjusted ORs (95% CIs) were obtained in conditional logistic regression models when adjusted for age, gender, place of residence, and smoking status.

§ *P* values calculated using *χ*^2^ tests.

The possible effect measure modification or effect modification of the association between TIMP2-418G/C SNP genotypes and CRC risk by various CRC risk factors including age, gender, and smoking status is summarized in Table 4. This involved studying the effect exerted on the association between the more common or wild genotype (GG) and the combined variant genotype (GC + CC) and the CRC risk by these various risk factors. On analyzing the effect modification of TIMP2-418G/C SNP genotypes by age, gender and smoking status, it was found that the effect of the combined variant genotype (GC + CC) on CRC risk was significantly dependent on gender (*P*=0.026). An increased CRC risk was observed in females [OR, 2.87 (95%CI, 1.04–7.90); *P*=0.041].

**Table 4:** Effect modification of TIMP2-418G/C SNP genotypes in presence of various risk factors of CRC in ethnic Kashmiri population

Genotype^*^ and characteristic	CRC cases *N* (%)^†^	Controls *N* (%)^†^	OR (95%CI); *P* value^‡^	Adjusted OR^§^ (95% CI); *P* value^‡^	*χ*^2^; Pearson *P* value (overall)^‡^^,^^║^
**Age**					
Wild and ≤50	58 (40.85)	67 (36.41)	**1.0 (Reference)**	**1.0 (Reference)**	
Variant and ≤50	8 (5.63)	24 (13.04)	**2.40 (1.00–5.73); 0.049**	**2.39 (0.99–5.73); 0.051**	6.68; 0.083
Wild and >50	59 (41.55)	64 (34.78)	1.09 (0.15–7.87); 0.929	1.12 (0.15–8.29); 0.913	
Variant and >50	17 (11.97)	29 (15.76)	1.68 (0.23–12.37); 0.608	1.72 (0.23–12.96); 0.599	
**Gender**					
Wild and male	67 (78.82)	72 (70.59)	**1.0 (Reference)**	**1.0 (Reference)**	
Variant and male	18 (21.18)	30 (29.41)	1.48 (0.75–2.91); 0.262	1.49 (0.74–3.00); 0.267	1.65; 0.199
Wild and female	50 (87.72)	59 (71.95)	**1.0 (Reference)**	**1.0 (Reference)**	
Variant and female	7 (12.28)	23 (28.05)	2.93 (1.07–8.02); 0.036	2.87 (1.04–7.90); 0.041	4.94; **0.026**
**Smoking status**					
Wild and non-smoker	52 (36.62)	64 (34.78)	**1.0 (Reference)**	**1.0 (Reference)**	
Variant and non-smoker	10 (7.04)	26 (14.13)	2.12(0.87–5.17); 0.101	2.13 (0.87–5.20); 0.096	6.5083; 0.089
Wild and smoker	65 (45.77)	67 (36.41)	0.93 (0.25–3.40); 0.910	0.92 (0.20–4.21); 0.911	
Variant and smoker	15 (10.56)	27 (14.67)	1.59 (0.39–6.52); 0.520	1.58 (0.32–7.77); 0.577	

* Wild refers to GG genotype and variant refers to GC + CC genotype.

^†^*N* denotes number of subjects or individuals.

‡ The values in bold indicate significant results. ORs (95% CIs) were obtained from conditional logistic regression models.

§ Adjusted ORs (95% CIs) were obtained from conditional logistic regression models when adjusted for age, gender, place of residence, and smoking status. The variable under consideration was excluded in the time of analysis.

║ *P* values calculated using *χ*^2^ tests.

The numbers and the frequencies of the subsets of various characteristics of the case group subjects under study i.e. age, gender, dwelling, smoking status, tumor location, tumor grade, and lymph node status for TIMP2-418G/C SNP are listed in Table 5. We analyzed the correlation of the TIMP2-418G/C promoter SNP with the subsets of these various characteristics of the case group subjects and none of these associations were found to be significant (*P*>0.05).

**Table 5:** Association of TIMP2-418G/C SNP with various clinico-pathological parameters, demographic variables, and environmental factors in CRC cases^*^

Characteristics	*N* = 142	GG 117(82.40%)	GC 25(17.60%)	CC 0(0%)	*χ*^2^; *P* value (overall)^*^
**Age (years)**					
≤50	66(46.48%)	58(49.57%)	8(32%)	0(0%)	2.557; 0.110
>50	76(53.52%)	59(50.43%)	17(68%)	0(0%)	
**Gender**					
Male	85(59.86%)	67(57.26%)	18(72%)	0(0%)	1.861; 0.173
Female	57(40.14%)	50(42.74%)	7(28%)	0(0%)	
**Dwelling**					
Rural	87(61.27%)	70(59.83%)	17(68%)	0(0%)	0.580; 0.447
Urban	55(38.73%)	47(40.17%)	8(32%)	0(0%)	
**Smoking status**					
Ever	80(56.34%)	65(55.56%)	15(60%)	0(0%)	0.165; 0.684
Never	62(43.66%)	52(44.44%)	10(40%)	0(0%)	
**Tumor location**					
Colon	58(40.85%)	47(40.17%)	11(44%)	0(0%)	0.125; 0.724
Rectum	84(59.15%)	70(59.83%)	14(56%)	0(0%)	
**Tumor grade**					
WD	95(66.90%)	81(69.23%)	14(56%)	0(0%)	1.628; 0.202
MD and PD	47(33.10%)	36(30.77%)	11(44%)	0(0%)	
**Lymph node status**					
Involved	78(54.93%)	63(53.85%)	15(60%)	0(0%)	0.315; 0.575
Not Involved	64(45.07%)	54(46.15%)	10(40%)	0(0%)	

* The values in bold indicate significant results. The abbreviations WD, MD, and PD denote well differentiated, moderately differentiated, and poorly differentiated, respectively.

### Association analysis of TIMP3-1296T/C (rs9619311) SNP

The frequencies of the genotypes of TIMP3-1296T/C SNP for both the case and the control groups are listed in Table 6. The more common TT genotype of TIMP3-1296T/C SNP was less frequent among the case group [43.66% (62/142)] in comparison with the control group [63.59% (117/184)]. The frequency of the heterozygous genotype (TC) in the case group [48.60% (69/142)] was higher than that of the control group [34.78% (64/184)]. The variant genotype (CC) also showed a higher frequency in the case group [7.74% (11/142)] in comparison with the control group [1.63% (3/184)]. Further, the frequency of the more common TIMP3-1296T allele was found to be 67.96% (193/284) among the case group subjects and 80.98% (298/368) among the control group subjects. The frequency of the less common TIMP3-1296C allele was found to be 32.04% (91/284) among the case group subjects and 19.02% (70/368) among the control group subjects (Table 6). The overall association between the TIMP3-1296T/C SNP and the modulation of colorectal cancer risk was found to be highly significant (*P*=0.000) (Table 6). The heterozygous genotype (TC) was significantly associated with a decreased risk of colorectal cancer [OR, 0.53 (95%CI, 0.32–0.86); *P*=0.011]. The variant genotype (CC) was also significantly associated with a decreased risk of colorectal cancer [OR, 0.18 (95%CI, 0.05–0.65); *P*=0.009]. Further, the less common TIMP3-1296C allele showed an overall significant association with colorectal cancer (*P*=0.0001). The TIMP3-1296C allele was associated with a decreased risk of colorectal cancer [OR, 0.50 (95%CI, 0.35–0.71); *P*=0.0002]. Also, the frequency of the combined variant genotype (TC + CC) consisting of heterozygous genotype (TC) and variant genotype (CC) grouped together was higher in the case group [56.34% (80/142)] in comparison with the control group [36.41% (67/184)]. The combined variant genotype (TC + CC) showed a strong overall significant association with colorectal cancer (*P*=0.000). The combined variant genotype (TC + CC) was significantly associated with a decreased risk of colorectal cancer [OR, 0.47 (95%CI, 0.29–0.75); *P*=0.002] (Table 6). Further, the genotype frequencies for TIMP3-1296T/C SNP among both the case and the control groups were found to be in agreement with HWE (cases: *χ*^2^ = 1.90; *P*=0.168 and controls: *χ*^2^ = 3.064; *P*=0.08).

**Table 6:** TIMP3-1296T/C SNP genotype frequency distributions among CRC cases and matched controls and risk of CRC^*^

	CRC cases (*N* = 142)^*^	Controls (*N* = 184)^*^	OR (95%CI); *P* value^†^	Adjusted OR^‡^ (95% CI); *P* value^†^	*χ*^2^; Pearson *P* value (overall)^†^^,^^§^
**Genotype**					
TT	62(43.66%)	117(63.59%)	**1.0 (Reference)**	**1.0 (Reference)**	16.52; **0.000**
TC	69(48.60%)	64(34.78%)	**0.53 (0.33–0.86); 0.010**	**0.53 (0.32–0.86); 0.011**	
CC	11(7.74%)	3(1.63%)	**0.18 (0.05–0.66); 0.009**	**0.18 (0.05–0.65); 0.009**	
TC + CC	80(56.34%)	67(36.41%)	**0.48 (0.30–0.76); 0.002**	**0.47 (0.29–0.75); 0.002**	12.85; **0.000**
**Allele**					
T	193(67.96%)	298(80.98%)	**1.0 (Reference)**		
C	91(32.04%)	70(19.02%)	**0.5 (0.35–0.71); 0.0002**		14.61; **0.0001**

* *N* denotes number of subjects or individuals.

† The values in bold indicate significant results. ORs (95% CIs) were obtained from conditional logistic regression models.

‡ Adjusted ORs (95% CIs) were obtained in conditional logistic regression models when adjusted for age, gender, place of residence, and smoking status.

§ *P* values calculated using *χ*^2^ tests.

The possible effect measure modification or effect modification of association between TIMP3-1296T/C SNP genotypes and CRC risk by various CRC risk factors including age, gender, and smoking status is summarized in Table 7. On analyzing the effect modification of TIMP3-1296T/C SNP genotypes by age, gender, and smoking status, it was found that the effect of the combined variant genotype (TC + CC) on CRC risk was significantly dependent on age (*P*=0.002). A decreased risk of CRC was observed in subjects with age ≤ 50 years [OR, 0.35 (95%CI, 0.17–0.71); *P*=0.003]. The effect of the combined variant genotype (TC + CC) on CRC risk was also significantly modified by gender (*P*=0.001). A decreased CRC risk was observed in females [OR, 0.30 (95%CI, 0.14–0.64); *P*=0.002]. Further, the effect of the combined variant genotype (TC + CC) on CRC risk was also significantly influenced by smoking status (*P*=0.004). A decreased CRC risk was observed in non-smokers [OR, 0.40 (95%CI, 0.20–0.79); *P*=0.009].

**Table 7:** Effect modification of TIMP3-1296T/C SNP genotypes in presence of various risk factors of CRC in ethnic Kashmiri population

Genotype^*^ and characteristic	CRC cases *N* (%)^†^	Controls *N* (%)^†^	OR (95%CI); *P* value^‡^	Adjusted OR^§^ (95% CI); *P* value^‡^	*χ*^2^; Pearson *P* value (overall)^‡^^,^^║^
**Age**					
Wild and ≤50	27 (19.01)	62 (33.70)	**1.0 (Reference)**	**1.0 (Reference)**	
Variant and ≤50	39 (27.46)	29 (15.76)	**0.34 (0.17–0.69); 0.003**	**0.35 (0.17–0.71); 0.003**	14.62; 0.002
Wild and >50	35 (24.65)	55 (29.89)	0.92 (0.12–6.84); 0.935	0.96 (0.13–7.31); 0.968	
Variant and >50	41 (28.87)	38 (20.65)	0.58 (0.08–4.40); 0.601	0.59 (0.076–4.61); 0.618	
**Gender**					
Wild and male	40 (47.06)	61 (59.80)	**1.0 (Reference)**	**1.0 (Reference)**	
Variant and male	45 (52.94)	41 (40.20)	0.638 (0.35–1.16); 0.139	0.71 (0.38–1.33); 0.287	3.03; 0.082
Wild and female	22 (38.60)	56 (68.29)	**1.0 (Reference)**	**1.0 (Reference)**	
Variant and female	35 (61.40)	26 (31.71)	**0.32 (0.15–0.67); 0.002**	**0.30 (0.14–0.64); 0.002**	12.04; 0.001
**Smoking status**					
Wild and non-smoker	28 (19.72)	61 (33.15)	**1.0 (Reference)**	**1.0 (Reference)**	
Variant and non-smoker	34 (23.94)	29 (15.76)	**0.41 (0.21–0.82); 0.011**	**0.40 (0.20–0.79); 0.009**	13.59; 0.004
Wild and smoker	34 (23.94)	56 (30.43)	0.66 (0.17–2.64); 0.561	0.87 (0.17–4.41); 0.862	
Variant and smoker	46 (32.39)	38 (20.65)	0.36 (0.09–1.42); 0.144	0.47 (0.10–2.30); 0.353	

* Wild refers to TT genotype and variant refers to TC + CC genotype.

^†^*N* denotes number of subjects or individuals.

‡ The values in bold indicate significant results. ORs (95% CIs) were obtained from conditional logistic regression models.

§ Adjusted ORs (95% CIs) were obtained from conditional logistic regression models when adjusted for age, gender, place of residence, and smoking status. The variable under consideration was excluded in the time of analysis.

║ *P* values calculated using *χ*^2^ tests.

The numbers and the frequencies of the subsets of various characteristics of the case group subjects under study i.e. age, gender, dwelling, smoking status, tumor location, tumor grade, and lymph node status for TIMP3-1296T/C SNP are listed in Table 8. We analyzed the correlation of the TIMP3-1296T/C promoter SNP with the subsets of these various characteristics of the case group subjects. The TIMP3-1296T/C SNP was significantly associated with gender (*P*=0.0130). Further, the male subjects who carried the variant genotype (CC) were at an increased risk of developing colorectal cancer in comparison with females [OR, 8.18 (95%CI, 1.62–41.28); *P*=0.0066]. The TIMP3-1296T/C SNP also showed an overall significant association with lymph node status (*P*=0.0117). Further, the subjects who carried the heterozygous genotype (TC) of TIMP3-1296T/C SNP were at a reduced risk of lymph node involvement [OR, 0.44 (95%CI, 0.22–0.89); *P*=0.0334]. Some statistical parameters including ORs mentioned here are not shown in Table 8 for the sake of simplification of data presentation.

**Table 8:** Association of TIMP3-1296T/C SNP with various clinico-pathological parameters, demographic variables, and environmental factors in CRC cases^*^

Characteristics	*N* = 142	TT 62(43.66%)	TC 69(48.60%)	CC 11(7.74%)	*χ*^2^; *P* value (overall)^*^
**Age(years)**					
≤50	66(46.48%)	27(43.55%)	37(53.62%)	2(18.18%)	5.17; 0.075
>50	76(53.52%)	35(56.45%)	32(46.38%)	9(81.82%)	
**Gender**					
Male	85(59.86%)	40(64.52%)	43(62.32%)	2(18.18%)	**8.69; 0.0130**
Female	57(40.14%)	22(35.48%)	26(37.68%)	9(81.82%)	
**Dwelling**					
Rural	87(61.27%)	39(62.90%)	39(56.52%)	9 (81.82%)	2.68; 0.262
Urban	55(38.73%)	23(37.10%)	30(43.48%)	2 (18.18%)	
**Smoking status**					
Ever	80(56.34%)	33(53.23%)	39(56.52%)	8(72.73%)	1.45; 0.48
Never	62(43.66%)	29(46.77%)	30(43.48%)	3(27.27%)	
**Tumor location**					
Colon	58(40.85%)	30(48.39%)	26(37.68%)	2(18.18%)	4.08; 0.13
Rectum	84(59.15%)	32(51.61%)	43(62.32%)	9(81.82%)	
**Tumor grade**					
WD	95(66.90%)	40(64.52%)	47(68.12%)	8(72.73%)	0.37; 0.83
MD and PD	47(33.10%)	22(35.48%)	22(31.88%)	3(27.27%)	
**Lymph node status**					
Involved	78(54.93%)	29(46.77%)	46(66.67%)	3(27.27%)	**8.9; 0.0117**
Not Involved	64(45.07%)	33(53.23%)	23(33.33%)	8(72.73%)	

* The values in bold indicate significant results. The abbreviations WD, MD, and PD denote well differentiated, moderately differentiated, and poorly differentiated, respectively.

## Discussion

In the present study, we investigated the role of functional TIMP2-418G/C and TIMP3-1296T/CSNPs in the promoter region of TIMP2 and TIMP3 genes, respectively, as a potential colorectal cancer risk factor in a case–control study design with 142 case subjects and 184 control subjects.

The TIMP2-418G/C SNP is located within and represents the third base of the consensus sequence (GAGGCTGGG) which is the binding site for zinc finger motif containing transcription factor, specificity protein-1/Sp1 [36,37]. The functional significance of this SNP is not fully known yet but it has been proposed that TIMP2-418 G to C transition abolishes the binding site of transcriptional activator, Sp1 and consequently the C allele may be associated with a markedly diminished promoter activity and reduced transcription which may result in decreased TIMP2 gene expression and eventually decreased TIMP2 activity and function including the inhibition of MMPs [36,37]. It is therefore possible that TIMP2-418G/C SNP induced down-regulation may alter TIMP MMP and non-MMP functions and disturb the physiological equilibrium between MMPs and TIMPs resulting in altered TIMP function including decreased inhibition and increased MMP activity which leads to altered ECM homeostasis and other physiological alterations which are critical events in neoplastic progression including that of CRC as discussed earlier [5,9,10,12]. The reduced TIMP2 expression has been reported in colorectal cancer [16].

The functional significance of TIMP3-1296T/C SNP involving T to C substitution at −1296 position is not known yet experimentally but through *in silico* analysis using TESS (URL: http://www.cbil.upenn.edu/tess), TRANSFAC (URL: http://thr.cit.nih.gov/molbio/signal/), and other online resources, it has been predicted to alter the transcription factor binding site [8,24]. Based on the *in silico* analysis, it is reasonable to postulate that the SNP induced alteration possibly influences the binding affinities of various transactivating nuclear proteins such that these proteins bind with higher affinity to the C allele resulting in higher TIMP3 gene expression in comparison with that of the expression in case of the T allele. Alternatively, the T to C transition represented by this SNP may be associated with decreased affinity of a putative transcription repressor protein to bind to its site on the TIMP3 promoter resulting again in a higher TIMP3 gene expression in case of the C allele in comparison with that of the expression in case of the T allele. However, these proposed functional effects need to be proved or supported by experimental evidence. The probable increase in promoter activity may result in enhanced transcription, which may result in increased TIMP3 gene expression and eventually increased TIMP3 activity and function including MMP inhibition. It is therefore possible that TIMP3-1296T/C SNP induced probable up-regulation may contribute to increased supply of TIMP3 protein to counter possible increase in MMP activity which otherwise leads to various diseases including cancers. As discussed earlier, the increased MMP activity is vital to neoplastic progression including that of CRC [5,9,10,12,38].

In the present study, we found that the heterozygous genotype (GC) of TIMP2-418G/C SNP was significantly associated with an increased risk of colorectal cancer. Further, the less common TIMP2-418C allele also showed a significant association with an increased risk of colorectal cancer. These findings are in agreement with several studies mentioned earlier that have reported that the GC and/or CC genotype of TIMP2-418G/C SNP is associated with a higher risk for the induction of various cancers and as discussed earlier this increased risk is mainly due to the altered physiological equilibrium between MMPs and TIMPs contributed to by this SNP. The TIMP2-418G/C SNP variant genotype (CC) was not found in either of the two study groups (case and control). This is due to the differences in the frequency and distribution of alleles in different world populations [39] and is possibly due to the genetic variability at multiple loci which are present widely across different ethnically defined populations throughout the world.

In our study, we found that the genotype frequencies of TIMP2-418G/C SNP among the control group subjects deviated from HWE. Many factors are known to be responsible for deviations from the HWE include non-random mating events (population stratification, inbreeding, and consanguinity), small population size, migration, mutations, and genotyping errors [40–42]. There can be multiple reasons for the deviations from HWE observed in our study. The population under study represents an almost pure ethnic population [43]. The population consists mostly of Muslims among whom consanguineous intra-familial marriages are quite common and often traditional [44]. Further, to a large extent the overall population in this region has remained relatively genetically isolated from the rest of the world which has resulted in a considerable degree of population stratification. The population is relatively small [43] and an important assumption that underlies the Hardy–Weinberg law is infinite population size and therefore small population size may be another contributory factor toward the HWE deviations observed in our study. The genotyping errors are also one of the main reasons for the deviation from HWE in association studies. However, it is unlikely that the deviations from HWE observed in our study are due to genotyping errors. This is because we obtained a high degree of the genotyping reproducibility of the samples for the SNPs under study and this is reflected by a weighted kappa coefficient of 0.99 obtained for this TIMP2 SNP in our study, which meant a high concordance rate of 99%.

In our study, we found a strong association of the TIMP3-1296T/C SNP with a decreased CRC risk across different allelic parameters. The heterozygous genotype (TC), variant genotype (CC), less common TIMP3-1296C allele and combined variant genotype (TC + CC) all were significantly associated with a decreased risk of colorectal cancer. These findings are in agreement with a study that associated this SNP with a decreased risk of HCC but in a gender-specific manner [32]. However, it is not in agreement with a study that reported the association of this SNP with an increased risk of breast cancer [24]. Few other studies found no association of this SNP with breast cancer [25], and bladder cancer [45]. It is important to mention here that the association studies of this SNP with various cancers is very limited and we could not find any report on its association with CRC. It is possible that the decreased CRC risk associated with this SNP in our population is possibly in part due to an increased TIMP3 expression which translates into increased inhibition of MMPs and proper state of physiological equilibrium between MMPs and TIMPs which is essential for maintenance of normal and disease free physiological environment. Further, TIMP3 has been reported to inhibit tumor growth, angiogenesis, invasion, and metastasis and promote apoptosis [46,47] and same has been reported *in vitro* in human colon cancer cell lines [22]. Further, decreased TIMP3 expression has been reported to correlate directly with an increased CRC invasion [20] and advanced tumor stage [21]. It is therefore reasonable to state that the increased TIMP3 expression and eventually the increased TIMP3 activity may be responsible for decreased CRC risk associated with this SNP in our population. However, it is important to mention here that the relationship between TIMP3 expression and cancer is not as straight as increased TIMP3 plasma levels have been associated with poor prognosis and decreased overall survival in oral cancer [48] and head and neck cancer [49]. However, as discussed earlier there is enough evidence for its anti-neoplastic role in CRC and our results are in agreement with those findings. It is reasonable to hypothesize here based on the findings of various studies that the role of TIMP3 can vary with different types of cancers and in different stages of same cancer. This can be largely attributed to diverse MMP-dependent and MMP-independent functions of TIMP3 and the influence of tumor microenvironment on its expression and activity. Therefore, studies involving comprehensive mechanistic evaluation of the role of TIMP3 in different cancers and in different stages of same cancer are warranted. Further, the functional evaluation of the SNP under study to decipher the actual role of this SNP in regulating TIMP3 expression and activity, in influencing the serum or circulating levels of TIMP3, and in modulating the susceptibility to various diseases including cancers such as CRC is warranted to arrive at a conclusive explanation.

In the present study, we also evaluated the possible effect modification of association between TIMP2-418G/C and TIMP3-1296T/C genotypes and CRC risk by age, gender, and smoking status. The reason for such an evaluation comes from the various studies that have shown that the susceptibility to CRC is significantly affected by age, gender, and smoking [50–52]. The effect measure modification or effect modification measures the variation of the outcome or an association between the primary exposure (here the SNP genotypes) and the disease or event under study (here CRC) due to the varying levels of a third factor (here age, gender and smoking status). In other words, the effect modification means that the association between primary exposure and disease is modified by varying levels of a third factor, often known as interaction factor or effect modifier. In our study, we found a significant effect modification of association between the combined variant genotype (GC + CC) of TIMP2-418G/C SNP and CRC risk by gender. The female gender increased the CRC risk. In other words, the females carrying the variant allele, TIMP2-418C in heterozygous form (GC) or variant homozygous form (CC) were at an increased risk of developing colorectal cancer in comparison with males. This finding can be partly explained by the studies, which have reported that the susceptibility to CRC is significantly affected by gender [52]. Generally, females have been reported to have a lower overall incidence of CRC than males [53]. The gender-specific differences in CRC susceptibility and prevalence possibly arise due to differences in levels of exposure to risk factors including occupational exposures, diet, or lifestyle and the possible gender-specific response to various risk factors. The hormonal differences between men and women also play a role. However, the disease risk and the disease prevalence are two different aspects of disease study. A particular gender may be more prone to a disease risk factor but the overall prevalence of the disease in that gender may be low in comparison with the other gender. This stands equally true for gene-based disease association studies including SNP analysis. Therefore, the overall prevalence of CRC may be lower in females in comparison with males but the modulation of risk by specific genotype of TIMP2-418G/C may result in the increased risk or susceptibility in females irrespective of the general prevalence status. Further, studies involving the mechanistic evaluation of the possible gender-specific differences in the expression and circulating levels of TIMP2 enzyme are warranted to obtain a conclusive answer.

In our study, we found a significant effect modification of association between the combined variant genotype (TC + CC) of TIMP3-1296T/C SNP and CRC risk by age, gender and smoking status. The age ≤ 50 years decreased the CRC risk. The study subjects aged ≤50 years and carrying the variant allele, TIMP3-1296C in the heterozygous form (TC) or variant homozygous form (CC) were at a decreased risk of developing colorectal cancer. The age is an independent CRC risk factor [52]. The likelihood of developing colorectal cancer increases sharply after 50 years of age [54]. The incidence rate of CRC increases progressively with age and approximately 90% of CRC incidence is reported in people aged 50 years or older. Further, the incidence rate of CRC in individuals aged more than 65 years has been reported to be more than 12 times higher in comparison with those with age less than 45 years [54]. Our finding that the study subjects aged ≤50 years were at a decreased risk of developing colorectal cancer is therefore in agreement with these various studies. Further, the female gender decreased the CRC risk. The females carrying the variant allele, TIMP3-1296C in heterozygous form (TC) or variant homozygous form (CC) were at a decreased risk of developing colorectal cancer in comparison with males. Our finding is in agreement with a study that reported the association of TC and CC genotypes of TIMP3-1296T/C SNP with a female-specific protective effect against HCC or in other words decreased risk of HCC in females in comparison with males [32]. The decreased CRC risk observed in the females in our study with respect to this SNP can be partly explained by the protective effect of female sex hormone estrogen [55] which has also been reported to exhibit protective effect against HCC [56]. This is opposite to the effect of male sex hormone testosterone which has been reported to promote cancer aggressiveness in males [57]. High TIMP3 expression associated with TC and CC genotypes coupled with the cancer protective function of estrogen may be responsible for the observed decreased CRC risk in females of our population when studied with respect to TIMP3-1296T/C SNP. However, it may be argued that the cancer-specific estrogen advantage diminishes in female subjects above the age of 55 years and cancer-specific testosterone disadvantage diminishes in male subjects above the age of 65 years when the secretion of estrogen and testosterone decreases considerably. But this valid argument seems insignificant in case of our study because almost half of the subjects in our study were ≤50 years of age and about half the number of female subjects were ≤50 years old. Those above 50 years were mostly in the 55–65 years range. Further, the non-smoking or never smoking decreased the CRC risk. The non/never-smoking study subjects carrying the variant allele, TIMP3-1296C in heterozygous form (TC) or variant homozygous form (CC) were at a decreased risk of developing colorectal cancer. Smoking is an established risk factor of CRC [50–52]. The smokers in comparison with non-smokers are exposed to increased burden of the carcinogenic agents from cigarette/tobacco smoking, which increases the risk of developing CRC in comparison with non-smokers. Thus, in response to our finding it is reasonable to argue that for same genotype or exposure level non-smokers compared with smokers are at a decreased risk of CRC.

In the present study, we also evaluated the association of TIMP2-418G/C and TIMP3-1296T/C SNPs with the numbers and the frequencies of the subsets of various characteristics of the case group subjects under study i.e. age, gender, dwelling, smoking status, tumor location, tumor grade, and lymph node status. However, we found no significant association between the subsets of any of these characteristics and the genotypic status of the TIMP2-418G/C SNP. In other words, we found no correlation between these characteristics and the modulation of CRC risk. Further, we found that the male subjects who carried the variant genotype (CC) of TIMP3-1296T/C exhibited a significantly higher risk of developing CRC in comparison with females. This finding is similar to the one observed in case of effect modification analysis. As already discussed, the decreased risk associated with females may be partly attributed to the cancer protective effect of the female sex hormone estrogen. However, further studies involving the evaluation of possible gender-specific differences in the expression and circulating levels of TIMP3 enzyme may provide a conclusive answer. In addition, the carriers of the heterozygous genotype (TC) of TIMP3-1296T/C SNP were at a reduced risk of lymph node infiltration. As discussed already, the heterozygous genotype (TC) and the variant genotype (CC) possibly exhibit relatively higher promoter activities and increased protein expression in comparison with the more common (TT) genotype. The decreased TIMP3 expression has been reported to correlate directly with increased CRC invasion [20] and advanced tumor stage [21]. Moreover, TIMP3 has been reported to inhibit tumor growth, angiogenesis, invasion, and metastasis and promote apoptosis [46,47]. It is therefore reasonable to hypothesize that the increased TIMP3 expression and eventually the increased TIMP3 activity possibly associated with TC and the CC genotypes may be responsible for decreased colorectal tumor invasion and metastasis, which also encompasses decreased lymph node infiltration. However, further studies involving comprehensive mechanistic evaluation of the expression and circulating levels of TIMP3 protein and the functional evaluation of the SNP under study to decipher the actual role of this SNP in regulating TIMP3 expression and activity is warranted to arrive at a conclusive explanation.

To the best of our knowledge, the present study is the first of its kind with regard to exploring the association of these SNPs with CRC risk in our population. The major strengths of the present study are the use of clinically diagnosed and histopathologically confirmed CRC samples, involvement of population-based controls in addition to the hospital-based controls which were recruited from the same geographical area during the same time period and were matched to the case group subjects individually for age, sex, place of residence (rural/urban), smoking habit and ethnicity in order to minimize the confounding effect of these relevant factors. Further, in our study, the results were adjusted for multiple potential confounding (third) variables. The major limitation of the present study is the modest sample size to detect comprehensively the gene–gene and gene–environment interactions, which usually require much larger sample size. Further studies incorporating a larger sample size and/or another ethnic population in our study are needed to substantiate our findings or confirm in depth the role of these SNPs in relation to CRC susceptibility. However, these limitations are unlikely to affect the outcome of the present study.

## Conclusions

We have demonstrated through the present study that the TIMP2-418G/C and TIMP3-1296T/C promoter SNPs modulate the CRC risk in ethnic Kashmiri population in a strong and highly significant manner. We have also shown that there is a significant effect modification of association between TIMP2-418G/C SNP genotypes and CRC risk by gender and of TIMP3-1296T/C SNP and CRC risk by age, gender, and smoking status. Further, we have also demonstrated that there is a significant association between TIMP3-1296T/C SNP and some characteristics of the case group subjects including gender and lymph node status.

## Abbreviations

CI: confidence interval
CRC: colorectal cancer
HWE: Hardy–Weinberg equilibrium
MMP: matrix metalloproteinase
OR: odds ratio
PCR-RFLP: polymerase chain reaction-restriction fragment length polymorphism
SNP: single nucleotide polymorphism
TIMP: tissue inhibitors of metalloproteinase
